# The ATF6 pathway of the ER stress response contributes to enhanced viability in glioblastoma

**DOI:** 10.18632/oncotarget.6712

**Published:** 2015-12-21

**Authors:** David Y.A. Dadey, Vaishali Kapoor, Arpine Khudanyan, Fumihiko Urano, Albert H. Kim, Dinesh Thotala, Dennis E. Hallahan

**Affiliations:** ^1^ Department of Radiation Oncology, Washington University School of Medicine, St. Louis, MO, USA; ^2^ Department of Medicine, Washington University School of Medicine, St. Louis, MO, USA; ^3^ Department of Pathology, Washington University School of Medicine, St. Louis, MO, USA; ^4^ Department of Neurological Surgery, Washington University School of Medicine, St. Louis, MO, USA; ^5^ Medical Scientist Training Program, Washington University School of Medicine, St. Louis, MO, USA; ^6^ Mallinckrodt Institute of Radiology, Washington University School of Medicine, St. Louis, MO, USA; ^7^ Siteman Cancer Center, Washington University School of Medicine, St. Louis, MO, USA; ^8^ Hope Center, Washington University School of Medicine, St. Louis, MO, USA

**Keywords:** ATF6, radioresistance, glioblastoma, ER-stress, GRP78

## Abstract

Therapeutic resistance is a major barrier to improvement of outcomes for patients with glioblastoma. The endoplasmic reticulum stress response (ERSR) has been identified as a contributor to chemoresistance in glioblastoma; however the contributions of the ERSR to radioresistance have not been characterized. In this study we found that radiation can induce ER stress and downstream signaling associated with the ERSR. Induction of ER stress appears to be linked to changes in ROS balance secondary to irradiation. Furthermore, we observed global induction of genes downstream of the ERSR in irradiated glioblastoma. Knockdown of ATF6, a regulator of the ERSR, was sufficient to enhance radiation induced cell death. Also, we found that activation of ATF6 contributes to the radiation-induced upregulation of glucose regulated protein 78 (GRP78) and *NOTCH1*. Our results reveal ATF6 as a potential therapeutic target to enhance the efficacy of radiation therapy.

## INTRODUCTION

Glioblastoma, the most common primary malignant brain tumor in adults, remains a diagnosis with dismal prognosis. Even with the adoption of surgical resection followed by concomitant chemo-radiation therapy and adjuvant chemotherapy as the current standard of care, the 5-year survival rate is 9.8% [1]. The poor outcome of glioblastoma is attributed to the highly infiltrative, proliferative and radioresistant nature of these tumors [2, 3]. The clinical relevance of radioresistance is evident in the failed attempts to improve patient outcomes by escalating radiation doses [4]. Radioresistance is thought to result from the numerous molecular aberrations that characterize glioblastoma, such as enhanced DNA damage repair (DDR) and activation of pro-survival signaling pathways [3, 5]. However some of these pathways, such as NF-κB, PI3K/Akt and cPLA_2_, have also been shown to be induced by ionizing radiation (IR) in various cell models [6-9]. In glioblastoma, induction of such pathways by IR may contribute to adaptive mechanisms that allow cells to survive radiation therapy, resulting in recurrent tumors.

A pathway of recent interest to many investigators, due to its role in adaptive survival signaling in cancer, is the endoplasmic reticulum stress response (ERSR) [10, 11]. The ERSR is a conserved cellular program that allows cells to cope with unfolded-protein-stress resulting from dysfunctions in cellular metabolism [12]. In normal cells, the ERSR is regulated by activating transcription factor 6 (ATF6), inositol-requiring protein 1 (IRE1) and protein kinase RNA-like ER kinase (PERK)[11]. These proteins are localized in the ER membrane and relay signals from the ER lumen to the cytosol and nucleus during ER stress [11]. Survival signaling downstream of ATF6, IRE1 and PERK converges on genes that promote ER-chaperone synthesis, ER-associated degradation (ERAD) and ER-membrane biogenesis [12]. Since tumor cells grow and spread rapidly, they often exhaust local blood supplies, resulting in areas of nutrient deprivation and hypoxia [13]. These adverse conditions perturb the sensitive protein folding environment in the ER, leading to ER stress [13]. Thus, in cancer cells, the ERSR is often deregulated and promotes tumorigenicity, as the deletion of tumor suppressors and/or activation of oncogenes favors cells that survive during high protein synthesis and metabolic stress [10, 13, 14]. Enhanced pro-survival signaling resulting from ERSR has been found to promote resistance to chemotherapy through upregulation of canonical targets such as glucose regulatory protein 78 (GRP78) [15, 16], and non-canonical targets such as Mcl-1 [17]. However the impact of ERSR-associated survival signaling on radioresistance in glioblastoma is unclear.

In previous work we identified GRP78 as a radiation inducible protein in models of glioblastoma and breast cancer [18]. Although GRP78 is a critical pro-survival chaperone involved in ERSR [16], upregulation of GRP78 by IR has not yet been shown to be associated with activation of ERSR. In this report, we show that IR can affect ER homeostasis and activate the ERSR. We demonstrate that activation of the ATF6 pathway in irradiated glioblastoma cells accounts for increased GRP78 levels, and that ATF6 contributes to radioresistance in these cells. We also identified Notch1 as a novel transcriptional target of ATF6, with a potential role in promoting an anti-apoptotic phenotype in irradiated glioblastoma [19, 20]. Our findings support the notion of targeting the ATF6 pathway as a potential strategy to improve the efficacy of radiation therapy for glioblastoma.

## RESULTS

### Ionizing radiation chronically alters cellular and ER redox homeostasis and is associated with ER membrane expansion

**Figure 1:**
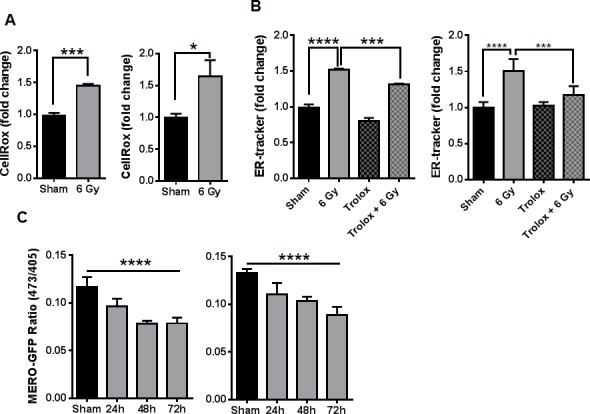
Ionizing radiation alters cellular redox and ER homeostasis in malignant gliomas **A.** Measurement of total ROS by flow cytometry of cells stained with CellRox Deep Red 48h after 6 Gy IR. **B.** The abundance of ER-tracker staining was assayed by flow cytometry 48h after 6 Gy IR in cells pretreated with DMSO or 50uM trolox for 3h. **C.** The change in GFP excitation peak was measured in cells transduced with MERO-GFP to determine ER-redox status 24, 48 and 72h after irradiation, shown is the ratio of fluorescence from excitation at 473nm and 405nm. In all graphs, data shown are the Means ±SD (*n* = 3). *P < 0.05, ***P < 0.001, ****P < 0.0001.

Previously we found that IR can enhance the binding of peptides that target GRP78 *in vivo* [18]*.* GRP78 is a well-characterized marker and regulator of ER stress. Since the biological effects of radiation are primarily mediated by oxidative stress [21], radiation-induced ROS might be associated with changes in ER homeostasis. We first examined the cellular ROS status of D54 and LN827 human glioblastoma cells 48h post irradiation (6 Gy) by using CellROX Deep Red Reagent and flow cytometric analysis. We found that irradiated D54 and LN827 had 45% [*P* < 0.001] and 78% [*P* < 0.05] elevations in ROS respectively (Figure 1A). We then evaluated the impact of IR on ER membrane expansion by using the ER-membrane-specific probe ER-Tracker Red. Flow cytometric analysis of D54 and LN827 cells stained with ER-Tracker showed a 52% and 50% increase in staining respectively 48h post IR [*P* < 0.0001], which was attenuated when cells were pre-treated with 50 uM Trolox for 2h prior to irradiation [*P* < 0.001] (Figure 1B). We measured the relative change in ER oxidative state by using lentiviral transduction to express an ER localized redox reporter, MERO-GFP, in D54 and LN827 cells. The fluorescence emission at 510 nm was measured by use of 473 nm and 405 nm excitation wavelengths, which represent reduced and oxidized MERO-GFP populations respectively [22]. In both cell lines, a progressive decrease in the 473/405 ratio was observed 24-72h after 6 Gy IR [D54: 0.07835 at 48h, *P* < 0.0001; LN827: 0.1032 at 48h, *P* < 0.0001] (Figure 1C). As an assay control, we used DTT to promote reduction of the ER lumen and found that treatment with 5mM DTT for 30 min was sufficient to increase the 473/405 ratio in both cell lines (Figure S1A). We isolated total cellular membranes from D54 and LN827 48h after irradiation and performed western blot analysis to examine GRP94 and GRP78 expression. We used VAPB as a loading control for the ER membrane, and observed that both GRP94 and GRP78 levels were increased in the membrane fraction 48h after irradiation (Figure S1B). These data suggest that radiation-induced oxidative stress triggers changes in ER homeostasis and may promote ER stress.

### Global induction of genes downstream of the ER stress response in irradiated glioblastoma cells

**Figure 2:**
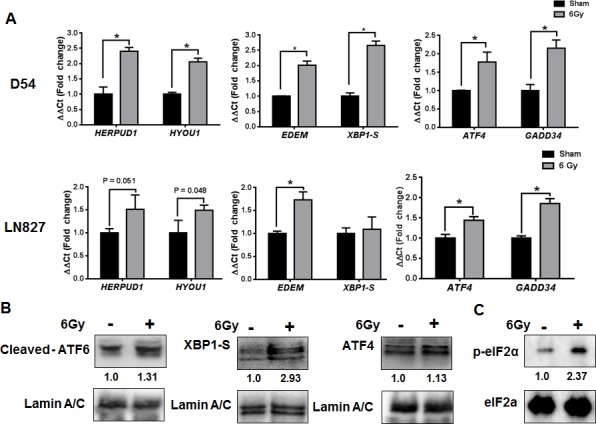
Global induction of genes downstream of the ER stress response in irradiated glioblastoma cells **A.** qRT-PCR analysis of genes downstream of ATF6, IRE1 and PERK using total-RNA extracts from D54 and LN827 cells 48h after 6 Gy IR. **B.** Western blot analysis of cleaved-ATF6, XBP1S and ATF4 in nuclear extracts from D54 cells harvested 48h after irradiation with 6 Gy. **C.** Western blot analysis of phosphorylated eIF2α in whole-cell lysates from D54 cells harvested 48h after irradiation with 6 Gy. Shown beneath the blots is the relative expression of the indicated proteins when compared to the respective untreated control. The values represent the normalized densitometric output for each band divided by the corresponding loading control. In all graphs, data shown are the Means ±SD (*n* = 3). *P < 0.05.

The observation of increased ER-chaperone expression, along with alterations in ER homeostasis, prompted us to examine the possibility that IR might be activating the ER stress response [23]. The ER stress response is regulated by three transmembrane proteins within the ER membrane: ATF6, IRE1 and PERK [11, 12]. To assay for activity of each pathway, we performed quantitative RT PCR (qRT-PCR) using primers specific for genes known to be downstream of each regulatory arm of the ERSR [23, 24]. For the ATF6 pathway, we assayed HERPUD1 and HYOU1 expression. For IRE1, we assayed EDEM and XBP1-S, and for PERK, we measured ATF4 and GADD34 levels. We found that IR (6 Gy) was associated with significant increases (*P* < 0.05) in expression of each of these genes in D54 and LN827 cells (Figure 2A). To validate our findings at the protein level, we assayed the expression of ATF6, XBP1-S and ATF4 in the nucleus of irradiated D54 cells. We found increased abundance of ATF6, XBP1-S and ATF4 48h after irradiation in nuclear fraction (Figure 2B). Furthermore, we examined the phosphorylation status of eIF2α, a proximal cytosolic target of activated PERK. We observed increased phosphorylation eIF2α 48h after irradiation (Figure 2C). Together, these findings support the notion that IR can promote mRNA and protein expression, which is consistent with activation of the ERSR in glioblastoma cells.

### Ionizing radiation activates the ATF6 pathway in glioblastoma

**Figure 3:**
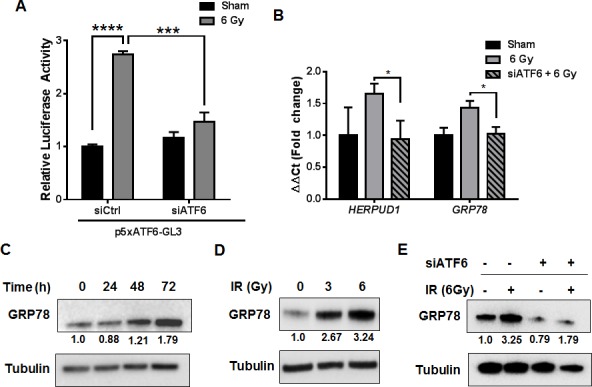
Activation of the ATF6 pathway in glioblastoma by ionizing radiation **A.** Luciferase assay for ATF6 transcriptional activity. D54 cells were co-transfected with p5xATF6-GL3 and siRNA targeting ATF6. The cells were irradiated with 6 Gy and assayed for luciferase activity 48h after IR. The relative activity normalized to control is shown. **B.** qRT-PCR analysis of *HERPUD1* and *GRP78* expression in D54 after knockdown of ATF6. Cells were treated with ATF6 siRNA for 48h prior to irradiation with 6 Gy, and harvested 48h later. **C.** Western blot analysis of GRP78 in whole-cell lysates from D54 cells harvested at the indicated time-points after irradiation with 6 Gy. **D.** Western blot analysis of GRP78 in whole-cell lysates from D54 cells harvested 48h after irradiation with 3 and 6 Gy. **E.** Western blot analysis of GRP78 in whole-cell lysates from D54 cells treated with ATF6 siRNA and 6 Gy IR. Cells were harvested 48h after irradiation. Shown beneath the blots is the relative expression of the indicated proteins when compared to the respective untreated control. The values represent the normalized densitometric output for each band divided by the corresponding loading control. In all graphs, data shown are the Means ±SD (*n* = 3). *P < 0.05, ***P < 0.001, ****P < 0.0001.

ATF6 is known to be the primary transcriptional regulator of several ER-chaperones, including GRP78 [25]. We determined if radiation can induce ATF6 transcriptional activity by co-transfecting D54 cells with p5xATF6-GL3 [26] and siRNA targeting ATF6. D54 cells were then treated with 6 Gy, and assayed for luciferase activity 48h after irradiation. We observed a 2.7 fold increase in luminescence in the irradiated cells [*P* < 0.0001] (Figure 3A). Irradiated cells that had been transfected with ATF6 siRNA had a blunted response, with a 1.4 fold increase in luminescence relative to the control siRNA group. The difference in luminescence between the irradiated control siRNA and ATF6 siRNA groups was statistically significant [*P* = 0.0002]. We observed elevations in GRP78 mRNA levels 48h after IR, where a 110% [*P* < 0.05] and 63% [*P* < 0.01] increase was found in D54 and LN827 respectively (Figure S2B). To test the requirement of ATF6 in mediating radiation induction of its target genes, we silenced ATF6 and assayed for HERPUD1 and GRP78 gene expression with qRT-PCR. We found that knockdown of ATF6 resulted in attenuated induction of ATF6 target genes, HERPUD1 and GRP78, in both D54 [*P* < 0.05] (Figure 3B) and LN827 [*P* < 0.01] (Figure S2C). We measured the abundance of GRP78 protein in the D54 glioblastoma cell line by western blot analysis at 24, 48 and 72h after a 6 Gy dose of x-radiation. We found that the levels of GRP78 protein were elevated at the 48 and 72h time points (Figure 3C). Knockdown of ATF6 was sufficient to abrogate GRP78 protein induction (Figure 3E). In order to study the potential relationship between radiation dose and induction of ATF6 activity, we treated D54 cells with 3 Gy and 6 Gy and analyzed GRP78 protein expression 48h after irradiation. We observed dose dependent increases in GRP78 levels (Figure 3D), which were reproducible in LN827 cells (Figure S2A). These results demonstrate that transcriptional activity of ATF6 is enhanced by IR, and that ATF6 is required for upregulation of GRP78 during the radiation response.

### Targeting ATF6 attenuates proliferation and clonogenic survival

**Figure 4:**
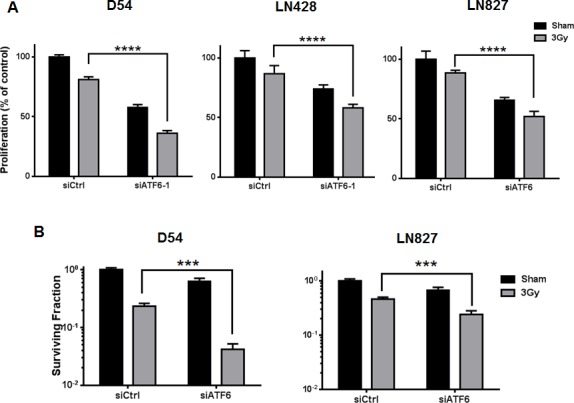
Knockdown of ATF6 attenuates cell proliferation and clonogenic survival in glioblastoma **A.** Proliferation assays of D54, LN428 and LN827. Cells were treated with siRNA targeting ATF6 for 48h prior to irradiation with 3 Gy, and proliferation was determined using a colorimetric cell proliferation assay 96h after irradiation. **B.** Clonogenic assays for D54 and LN428. Cells were treated with ATF6 siRNA for 48h and re-seeded at low density prior to irradiation with 3 Gy. After 7-10 days, colonies comprised of 50 or more cells were scored. In all graphs, data shown are the Means ±SD (*n* = 5). **P < 0.01, ***P < 0.001, ****P < 0.0001.

Through pro-survival signaling mediated by ATF6, activation of the ER stress response has been shown to contribute to enhanced viability in several cancers [10, 27]. Thus, we hypothesized that the activation of ATF6 by radiation-induced ER stress could play a role in promoting the viability of irradiated glioblastoma. To test this hypothesis, we silenced ATF6 and studied proliferation and clonogenic survival. Proliferation assays were performed after transiently transfecting D54, LN428 and LN827 with ATF6-specific siRNA prior to irradiation (3 Gy), and allowed to proliferate for 96h. Persistent knockdown of ATF6 after 96h was verified by western blot analysis (Figure S2D). We found that in D54, LN428 and LN827 cell lines, silencing ATF6 alone was sufficient to attenuate cell proliferation (Figure 4A). Furthermore when ATF6 knockdown was combined with 3 Gy irradiation, we observed significantly reduced cell proliferation when compared to the irradiated non-targeting siRNA (siCtrl) group [Mean difference: 45% in D54, *P* < 0.0001; 23% in LN428 *P* = 0.0015; 36% in LN827, *P* < 0.0001] (Figure 4A). To rule out the potential off target effects of this siRNA, we used an additional siRNA and shRNA targeting ATF6. We found that the effect of these different RNAi approaches was consistent with our primary results (Figure S3B and S3D). Similarly, colony formation was ascertained in D54 and LN428 cells following siRNA treatment and 3 Gy. In D54, the combination of radiation and ATF6-knockdown resulted in a 10 fold decrease in mean surviving fraction when compared to the siCtrl group [*P* < 0.001] (Figure 4B). Similar results were found in LN428, where silencing ATF6 prior to radiation lead to a 1.9 fold decrease in mean surviving fraction [*P* < 0.001](Figure 4B). In order to examine the impact of increasing radiation dose on clonogenic survival in the context of ATF6-knockdown, we studied clonogenic survival using 0, 2, 4 and 6 Gy IR doses in D54 after treatment with ATF6 siRNA. Significant reduction in clonogenic survival was observed after targeting ATF6 in D54 cells [Mean surviving fraction: 23%, 1.2% and 0.21% at 2, 4 and 6 Gy respectively; *P* < 0.05] (Figure S3A). Further we investigated the effect of ATF6 knockdown on cellular ROS levels. We found that knockdown of ATF6 alone with two different siRNA constructs resulted in 1.2 and 1.3 fold increases in cellular ROS respectively (Figure S4). When combined with irradiation, ATF6 knockdown with two different siRNA constructs lead to a 1.5 and 1.7 fold increase in cellular ROS respectively (Figure S4). These findings suggest that targeting ATF6 can enhance the efficacy of IR, and that the effect of ATF6 knockdown may involve altered regulation of cellular ROS.

### Knockdown of ATF6 enhances radiation induced cell death in glioblastoma

**Figure 5:**
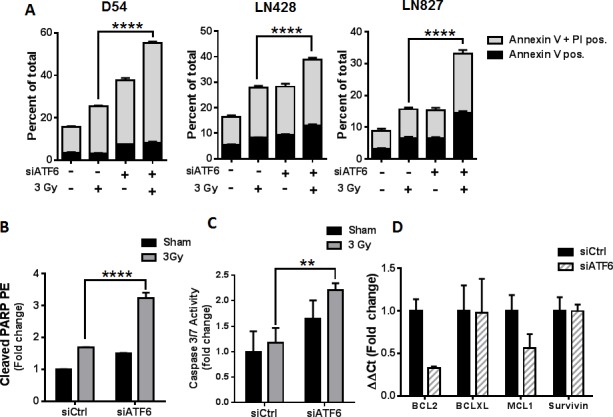
Knockdown of ATF6 enhances radiation-induced cell death in glioblastoma **A.** Annexin V/PI assays of D54, LN428 and LN827. Cells were treated with siRNA targeting ATF6 for 48h prior to irradiation with 3 Gy. Cells were stained with Annexin V and PI 96h after irradiation, and analyzed by flow cytometry. **B.** Cleaved PARP assay in D54. Cells were treated with ATF6 siRNA for 48h prior to irradiation with 3 Gy, and analyzed 96h later by flow cytometry after staining with anti-cleaved PARP-PE antibody. **C.** Caspase 3/7 activity assay in D54. Cells were treated with ATF6 siRNA for 48h prior to irradiation with 3 Gy, and analyzed 48h later by micro-plate reader **D.** qRT-PCR analysis of *BCL2, BCL-XL*, *MCL1*, and *Survivin* gene expression in D54 after knockdown of ATF6. In all graphs, data shown are the Means ±SD (*n* = 3). **** *P* < 0.0001.

We began our characterization of the mode of cell death induced by ATF6 knockdown by examining apoptotic cell death with Annexin-V/PI assays. D54, LN428 and LN827 Cells were treated with ATF6 siRNA and 3 Gy, and stained with Annexin-V and PI 96h later. Flow cytometric analysis of the cells showed that ATF6 knockdown resulted in an increase in early apoptosis (Annexin-V positive) and late apoptosis (Annexin-V + PI positive). The mean percent of total population was 55.3% (*P* < 0.0001), 38.7% (*P* < 0.0001), and 33.1% (*P* < 0.0001) in D54, LN428 and LN827 respectively (Figure 5A). Similar results were observed when targeted with another siRNA targeting ATF6 (Figure S5). ATF6 knockdown enhanced PARP cleavage by 3.2 fold in D54, as detected by flow cytometry 96h after irradiation with 3 Gy (*P* < 0.0001) (Figure 5B). We also examined caspase 3/7 activity in D54 after ATF6 knockdown and irradiation. Knockdown of ATF6 in D54, when combined with irradiation, resulted in a 2.2 fold increase in caspase 3/7 activity (Figure 5C). Furthermore, qRT-PCR of D54 cells 48h after ATF6 siRNA treatment revealed a 67% and 44% downregulation of BCL2 and MCL1 gene expression respectively (Figure 5D). These data indicate that ATF6 knockdown sensitizes glioblastoma cells to radiation-induced apoptosis.

### ATF6 contributes to regulation of NOTCH1 gene expression

**Figure 6:**
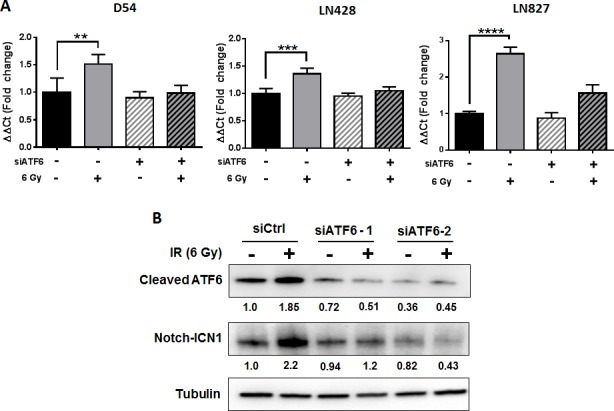
ATF6 contributes to regulation of NOTCH1 gene expression **A.** qRT-PCR analysis of *NOTCH1* expression in D54, LN428 and LN827. Cells were treated with siRNA targeting ATF6 for 48h prior to irradiation with 6 Gy. All qRT-PCR analyses were performed using cells harvested 48h after irradiation. **B.** Western blot analysis of cleaved-ATF6 and Notch-ICN1 in whole cell lysates of D54 harvested 48h after treatment with ATF6 siRNA and irradiation with 6 Gy. Shown beneath the blots is the relative expression of the indicated proteins when compared to the respective untreated control. The values represent the normalized densitometric output for each band divided by the corresponding loading control. In all graphs, data shown are the Means ±SD (*n* = 4). **P < 0.01, ***P < 0.001, **** *P* < 0.0001.

The mechanisms by which ATF6 may be linked to anti-apoptotic signaling are largely defined by its role in regulation of the GRPs [10], of which GRP78 is known to suppress caspase-7 activity [16, 28, 29] and potentiate survival signaling through Akt [30, 31]. Recent studies have demonstrated that the antiapoptotic transcriptome in irradiated glioblastoma is widely altered, with several genes having unknown upstream links to radiation response [20]. Since our results support the notion that ATF6 is a radiation-responsive transcription factor, we hypothesized that ATF6 contributes to the regulation of antiapoptotic genes after irradiation. We screened radiation-responsive antiapoptotic genes [20] for putative ATF6 binding sites [26] and identified *BLC6*, *BTG2*, *HMGB2* and *NOTCH1* as potential targets of ATF6. We used qRT-PCR to study gene expression of *BLC6*, *BTG2*, *HMGB2* and *NOTCH1* in D54 cells treated with ATF6 siRNA. We found that *NOTCH1* was induced by 51% (*P* < 0.01) at 48h after irradiation, and that this induction was abrogated by ATF6 knockdown (Figure 6A). The contribution of ATF6 to radiation-induction of *NOTCH1* was also evident in LN428 and LN827 cell lines, where knockdown of ATF6 attenuated radiation induction of *NOTCH1* gene expression from 35% to 4% in LN428, and 164% to 56% in LN827 (Figure 6A).

To validate these findings at the protein level, we performed western blot analysis of cleaved-ATF6 and Notch-ICN1 in D54 cells treated with siRNAs targeting ATF6. We found that while irradiation was associated with increased abundance of cleaved-ATF6 and Notch-ICN1, knockdown of ATF6 with two different siRNAs was sufficient to abrogate induction of both cleaved-ATF6 and Notch-ICN1 (Figure 6B). To determine the effect of Notch1 knockdown on radiation resistance, we evaluated cell proliferation after knockdown of Notch1. We found that knockdown of Notch1 alone reduced cell proliferation by 18% (Figure S3B). When Notch1 knockdown was combined with irradiation, we observed that cell proliferation was attenuated by 59% (Figure S3B). In summary, these results demonstrate that ATF6 may be involved in regulating *NOTCH1* mRNA and protein expression, and that Notch1 may play a role in promoting proliferation of irradiated glioblastoma cells.

## DISCUSSION

This study investigated mechanisms by which IR could trigger stress responses in glioblastoma, and determined if such stress responses could impact cell survival. In this study, we found that by activating the ERSR, IR can contribute to adaptive survival signaling in glioblastoma through a mechanism that involves regulation of ATF6.

In our first set of experiments, we studied a potential link between irradiation and ER homeostasis in glioblastoma cell lines. The ER is known to be responsive to numerous forms of biochemical perturbation, including calcium balance, metabolism and reduction/oxidation [11]. IR has been shown to primarily alter ROS balance in cells by triggering hydrolysis of water molecules, thereby generating hydroxyl radicals [32]. Furthermore, there have been reports that ROS can activate ER stress signaling in gastric and colorectal cancer cell lines [33, 34]. Thus, we hypothesized that IR could alter ER redox balance in a manner that could promote ER stress. This hypothesis was supported by our observation that persistent elevations in cellular ROS occurred in our cell lines after irradiation (Figure 1A), and our measurement of an increased oxidative state specifically within the ER lumen (Figure 1C). In order to examine whether these changes in cellular and ER ROS coincided with gross alteration in ER homeostasis, we investigated changes in ER-membrane abundance and levels of GRP78 and GRP94 ER chaperones. Expansion of the ER membrane is known to occur during ER stress, as the ERSR drives synthesis of ER-membrane lipids and chaperones in order to expand the protein folding capacity of the ER [12]. We found expansion of the ER-membrane after irradiation, and pre-treatment with a ROS scavenger was sufficient to attenuate this expansion (Figure 1B). Similarly, we observed increased abundance of both GRP78 and GRP94 (Figure S1B). Together, these results suggest that there is a link between ROS generated by IR and changes in ER-homeostasis in glioblastoma. A mechanism by which IR-induced ROS can promote ER stress remains to be shown. We postulate that lipid peroxidation secondary to IR could affect the integrity of the ER membrane, thereby affecting transport processes associated with ER homeostasis. Alternatively, IR could be activating a secondary process that may lead to gradual accumulation of ROS in the ER. This is supported by our observation that ER-oxidation increases gradually over several days (Figure 1C).

The hypothesis that IR can induce the ERSR in glioblastoma was further supported by the observation of global activation of gene targets downstream of each arm of the ERSR: ATF6, IRE1 and PERK (Figure 2). Induction of ERSR signaling by IR occurs in endothelial cells [35, 36] and IEC-6 intestinal epithelial cells [37]; however these studies used doses of radiation upwards of 15 Gy. IR induced ERSR signaling in cancer is supported by IR induction of the PERK/eIF2a pathway [38, 39]. The PERK pathway is often considered to be the switch in the ERSR that regulates ER stress-induced cell death [40], and is known to transiently promote survival during ER stress [12]. Our findings suggest that the ATF6 and IRE1 pathways are also activated in irradiated glioblastoma cell lines. These pathways are primarily associated with the pro-survival aspects of the ERSR and may represent a cellular mechanism by which glioblastoma could adapt to therapeutic stress.

We further studied the ATF6 arm of the ERSR because of its well established role as the primary regulator of pro-survival chaperone synthesis, and also because of its potential role as a radiation-responsive transcription factor. Not only did we find that ATF6 transcriptional activity was enhanced in irradiated glioblastoma (Figure 3A), but we also found accumulation of GRP78, a major target of ATF6, in a time and dose-dependent manner (Figure 3C and 3D). Additionally, ATF6 was required for radiation induction of GRP78 (Figure 3B and 3E). Enhanced expression of GRP78 in glioblastoma has been associated with increased resistance to temozolomide, cisplatin and etoposide [28, 29], with proposed mechanisms involving suppression of caspase-7 activation [41]. Silencing GRP78 in glioblastoma has been shown to increase sensitivity to gamma-irradiation [28]. Furthermore, overexpression of GRP78 has been observed in patient-derived recurrent-glioblastoma specimens, and has been shown to inversely correlate with progression-free survival [42]. In the context of these studies, our findings suggest that radiation induced ERSR signaling contributes to an adaptive pro-survival phenotype in glioblastoma that could result in therapeutic resistance and tumor recurrence.

Given the potential for ATF6 activation to mediate pro-survival signaling within glioblastoma, we silenced ATF6 and measured cell sensitivity to IR. We found that silencing ATF6 was sufficient to enhance the cytotoxic effects of radiation in glioblastoma (Figure 4). The enhanced cell death represented in the clonogenic survival assays is likely due to potentiation of apoptotic signaling during ATF6 knockdown (Figure 5). However, it is possible that the mechanism of cell death may be more complex, as recent literature suggests that necroptosis is also activated by ER stress [43, 44]. Additional investigations are needed to fully characterize the cell death mechanisms that may be downstream of ER stress and ATF6 knockdown in GBM. We have also found that ATF6 knockdown results in altered cellular ROS regulation and enhances the induction of ROS in irradiated cells (Figure S4). However it remains to be shown how ATF6 is involved in cellular ROS balance, and whether deregulation of this process may contribute to increased radiosensitivity. We hypothesize that the connection between loss of ATF6 and reduced survival might be explained by the downstream reduction of pro-survival GRPs secondary to ATF6 knockdown.

While ATF6 has a well characterized role in regulation of ER-stress chaperone synthesis, a search for putative ATF6 binding sites (unfolded protein response elements - UPRE) in the eukaryotic promoter database reveals many genes thought to be unrelated to ER stress [26, 45, 46]. This prompted us to examine the possibility that ATF6 could be regulating other anti-apoptotic gene targets. Transcriptomic analysis of irradiated glioblastoma identified a number of anti-apoptotic genes with consistently altered gene expression [20]. By screening this list of genes for putative UPRE sequences, we identified *BCL6*, *NOTCH1*, *HMGB2* and *BTG2* as potential targets of ATF6. In our qRT-PCR analyses, we found that ATF6 contributed to *NOTCH1* upregulation after irradiation (Figure 6), but was dispensable for *BCL6*, *HMGB2* and *BTG2* expression (Figure S6). The notch pathway is critical to the differentiation of neural progenitor cells during normal brain development, and has also been linked to cellular responses to hypoxia and angiogenesis [47]. Notch1, through regulation of PI3K/Akt activity and Mcl-1, has been implicated in the radioresistance of glioma initiating cells [19, 48]. Additionally, increased Notch1 expression has been demonstrated in recurrent glioblastomas after chemo-radiation therapy and was identified as a prognostic marker for anti-angiogenic therapy [49]. *NOTCH1* expression could be, in part, a product of ERSR signaling downstream of ATF6 in the context of radiation response.

In summary, we found that radiation can induce ER stress signaling in a manner that contributes to viability of glioblastoma cells. Specifically, we identified the ATF6 pathway of the ER stress response as a radiation responsive signaling pathway, and *NOTCH1* as a previously unidentified target of ATF6 with a potential role in mediating survival of glioblastoma cells during the radiation response. While additional work is needed to validate the mechanisms by which Notch1 may be involved in promoting cell survival downstream of ATF6, this investigation supports other studies identifying Notch1 as a key component in therapeutic resistance in glioblastoma. Furthermore, this work highlights the potential for a new therapeutic strategy wherein targeting of the ERSR through inhibition of ATF6 may serve to enhance the efficacy of radiation therapy for glioblastoma.

## MATERIALS AND METHODS

### Cell cultures and chemicals

The D54 cell line [50] was a generous gift from Dr. Yancey Gellipsie. LN428 and LN827 [51]cell lines were gifts from Dr. Joshua Rubin. 293T cells were obtained from ATCC. D54 was maintained in DMEM with F-12 Nutrient Mixture in a 1:1 ratio, 10% fetal bovine serum (FBS) and 1% penicillin-streptomycin (P/S). LN428 and LN827 were maintained in DMEM with 10% FBS and 1% P/S. 293T cells were maintained in DMEM low-glucose with 10% FBS and 1% P/S. All cultures were grown at 37°C in a humidified atmosphere with 5% CO_2_. Trolox, 2-deoxy-glucose, and thapsigargin were purchased from Sigma (St. Louis, MO, USA). Radiation was administered to cells at a dose rate of 2.5Gy/min by means of a RS2000 160kV X-ray Irradiator with a 0.3 mm copper filter (Rad Source Technologies, Suwanee, GA, USA).

### Lentiviral transduction

pLenti-MERO-GFP plasmid was a kind gift from Dr. Fumihiko Urano. Lentiviruses were constructed by co-transfecting pLenti-MERO-GFP, the packaging plasmid (pCMV-dR8.2), and the envelope plasmid (pCMV-VSV-G) into 293T cells using Fugene 6 (Roche, Mannheim Germany). Transfections were carried out for 18h, after which the culture medium was changed. Viral supernatants were harvested 48h after transfection, and used to infect 100,000 target cells in the presence of 10 ug/ml polybrene. Cells were incubated with virus supernatant for 18h, after which they were allowed to recover for 24h before selection with 2 ug/ml puromycin for 72h. Puromycin-resistant cells were then used in downstream assays.

### Quantitative real-time PCR analysis

RNA was extracted with TRIZOL reagent (Life Technologies, Foster City, CA, USA) and isolated with RNeasy Mini Kit (Qiagen, Germantown, MD, USA). 1 μg of RNA was used to produce cDNA with High Capacity cDNA Reverse Transcrptase Kit (Applied Biosystems, Foster City, CA, USA). Primer sequences were obtained from the Primer Bank [52-54] (Supplementary Table 1.) and were used in quantitative real-time PCR (qRT-PCR) with a 7900HT Real-Time PCR System (Applied Biosystems). The delta-delta Ct method for quantitation of relative gene expression was used to determine the mean expression of each target gene normalized to the geometric mean of actin and GAPDH.

### Measurement of ER oxidation status

ROS levels in the ER were measured in D54 and LN827 cells transduced with MERO-GFP. Cells were irradiated with 6 Gy, and trypsinized at the expirmental end-point. 15,000 viable cells were added to each well of a 96-well plate, after which the fluorescence at 510 nm was measured from the 405 nm and 473 nm excitation wavelengths with a SpectraMax i3 microplate reader (Molecular Devices, Sunnyvale, CA, USA). The 473/405 nm fluorescence ratio was then calculated for each treatment group.

### Flow cytometry

#### i) Cellular ROS studies

D54 and LN827 cells were treated with or without radiation. At the experimental endpoint, 5 uM CellROX Deep Red Reagent (Life Technologies) was added to cell culture media and incubated for 30 min at 37°C. Cells were washed with PBS, collected, and analyzed by flow-cytometry with a MACSQuant Analyzer (Miltenyi Biotec, San Diego, CA, USA).

#### ii) ER tracker studies

100,000 D54 and LN827 cells were seeded in 6-well plates and treated with 50 uM trolox for 3h prior to irradiation with 6 Gy. Cells were washed twice with PBS, and complete medium was added to the wells. 48h after treatment, media was changed to 1 uM ER-Tracker Red (Life Technologies) in HBSS and left to incubate for 30 min at 37°C. The cells were then washed twice with fresh HBSS, collected, and analyzed by flow cytometry.

#### iii) Annexin V/PI studies

D54, LN827 and LN428 cells were transfected with siRNA and re-seeded in 6-well plates for radiation treatments. 96h after irradiation with 3 Gy, the cells were collected and stained with Annexin V-FITC and PI (BD Biosciences, San Jose, CA, USA) according to the manufacturer's protocol. Stained cells were analyzed by flow cytometry.

### Luciferase assays

Plasmids encoding p5xATF6-GL3 was a gift from Dr. Ron Prywes (Addgene plasmid #11976). D54 cells grown in 60 mm plates were transfected with 2 ug of p5xATF6-GL3 using Fugene 6 according to the manufacturer's protocol. After transfecting for 18h, cells were re-plated and allowed to adhere prior to irradiation with 6 Gy IR. 48h after IR, the cells were lysed and assayed for luciferase activity using Luciferase Assay System (Promega, Madison, WI, USA) and a microplate reader.

### Capsase activity assay

Cells were seeded in 96-well plates after transfection with siRNA. Cells were irradiated with 3 Gy and Caspase 3/7 activity was measured using Caspase-Glo assay kit (Promega, Madison USA) according to the manufacturer's protocol.

### Western immunoblot analysis

M-PER mammalian protein extraction reagent (Thermo-Pierce, Rockford, IL, USA) was used to extract total soluble protein from cells. For extraction of cellular membrane proteins, the Proteo Extract Subcellular Proteome Extraction Kit (Millipore, Lake Placid, NY, USA) was used according to the manufacturer's protocol. For extraction of nuclear proteins, NE-PER Nuclear and Cytoplasmic Extraction Reagents were used (Thermo). Concentrations of cell lysates were determined by BCA assays, and equal amounts of protein were resolved by SDS-PAGE and transferred to PVDF membranes for immunoblotting. GRP78 and VAPB antibodies were from ProteinTech (Chicago, IL, USA). Notch1 and GAPDH antibodies were from Cell Signaling Technology (Danvers, MA, USA). Tubulin antibody was from Sigma. Blots were imaged with a ChemiDoc-MP Imaging System (Bio-Rad Laboratories, Hercules, CA, USA), and analyzed with Image Lab Software (Bio-Rad).

### Transfection of siRNA

Silencer-select pre-designed siRNAs against ATF6 (s223544 and s22688), NOTCH1 (s9634) and non-targeting control siRNA were purchased from Life Technologies/Ambion. Lipofectamine RNAiMax transfection reagent (Life Technologies/Ambion) was used to deliver siRNAs according to manufacturer's protocol. Gene silencing was confirmed 48h after transfection by qRT-PCR.

### Clonogenic survival assays

Cells were seeded at defined cell densities according to radiation dose and allowed to attach overnight. Cells were then irradiated with 0, 2, 3, 4, or 6 Gy. After incubating for 7-10 days, plates were stained with 0.5% crystal violet. Colonies comprised of 50 cells or more were counted and the counts were normalized to plating efficiency and represented as surviving fraction relative to control (sham/non-targetting siRNA).

### Cell proliferation assays

3000 cells per/well were seeded in 96 well plates 24h after siRNA transfection. Cells were irradiated with 3 Gy and allowed to grow for 96 h. Proliferation was determined by adding 10 μL of PrestoBlue cell viability reagent (Life Technologies) to each well. After incubating at 37°C for 15 min, the fluorescence was measured at 560^ex^/590^em^ with a microplate reader.

### Statistical analysis

Where indicated, statistical analyses were performed using the Student's *t* test and one-way or two-way analysis of variance (ANOVA). Bonferroni's multiple comparisons test was applied where necessary. These analyses were performed in Prism 6 (GraphPad Software, La Jolla, CA, USA), and statistical significance was indicated on each graph where appropriate.

## SUPPLEMENTARY MATERIAL FIGURES AND TABLE


