# Supplementary material for: The ATF6 pathway of the ER stress response contributes to enhanced viability in glioblastoma
Source: Oncotarget. 2015 Dec 21;7(2):2080–92. doi: 10.18632/oncotarget.6712 (PMC4811517; doi:10.18632/oncotarget.6712)
Supplement: Supplementary file 1 [file oncotarget-07-2080-s001.pdf]

The ATF6 pathway of the ER stress response contributes to enhanced viability in glioblastoma

Supplementary Material

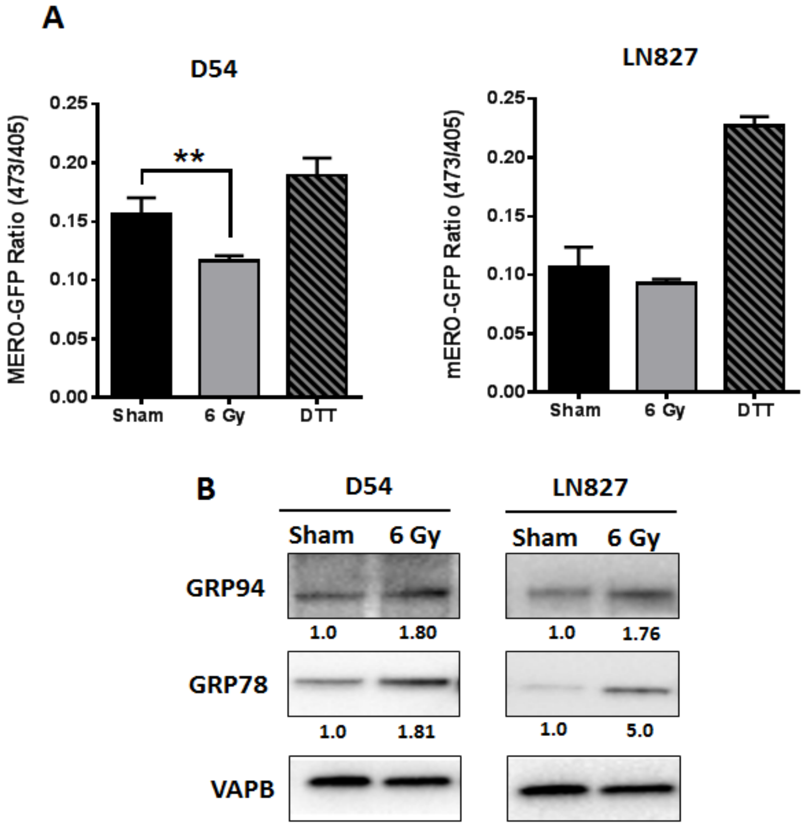

Supplementary Figure 1: Evaluation of MERO-GFP with DTT treatment and expression of ER chaperones in membrane extracts. (A) The change in GFP excitation peak was measured in cells transduced with MERO-GFP to determine ER-redox status 48h after irradiation and 30min after treatment with 5mM DTT, shown is the ratio of fluorescence from excitation at 473nm and 405nm. (B) Western blot analysis of GRP78 and GRP94 expression in cellular membrane extracts from D54 and LN827 48h after 6 Gy IR, VAPB was used as a loading control for the membrane fraction. In all graphs, data shown are the Means ±SD (n = 3).

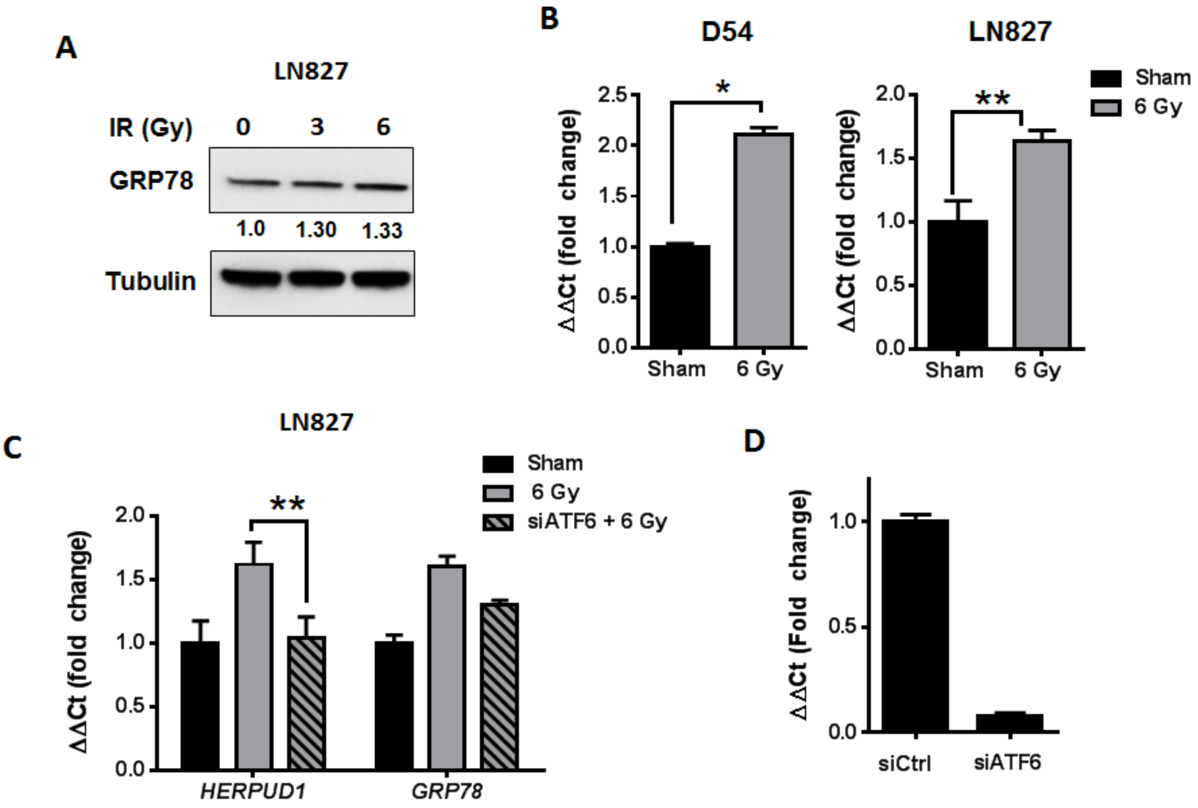

Supplementary Figure 2: Induction of GRP78 and HERPUD1 mRNA, and validation of ATF6 knockdown. (A) Western blot analysis of GRP78 in whole-cell lysates from LN827 cells harvested 48h after irradiation with 3 and 6 Gy. (B) qRT-PCR analysis of *GRP78* expression in D54 and LN827 cells harvested 48h after irradiation with 6 Gy. (C) qRT-PCR analysis of *HERPUD1* and *GRP78* expression in LN827 after knockdown of ATF6. Cells were treated with ATF6 siRNA for 48h prior to irradiation with 6 Gy, and harvested 48h later. (D) qRT-PCR analysis of ATF6 gene expression in D54 cells 48h after transfection with ATF6 siRNA. In all graphs, data shown are the Means ±SD (n = 3).

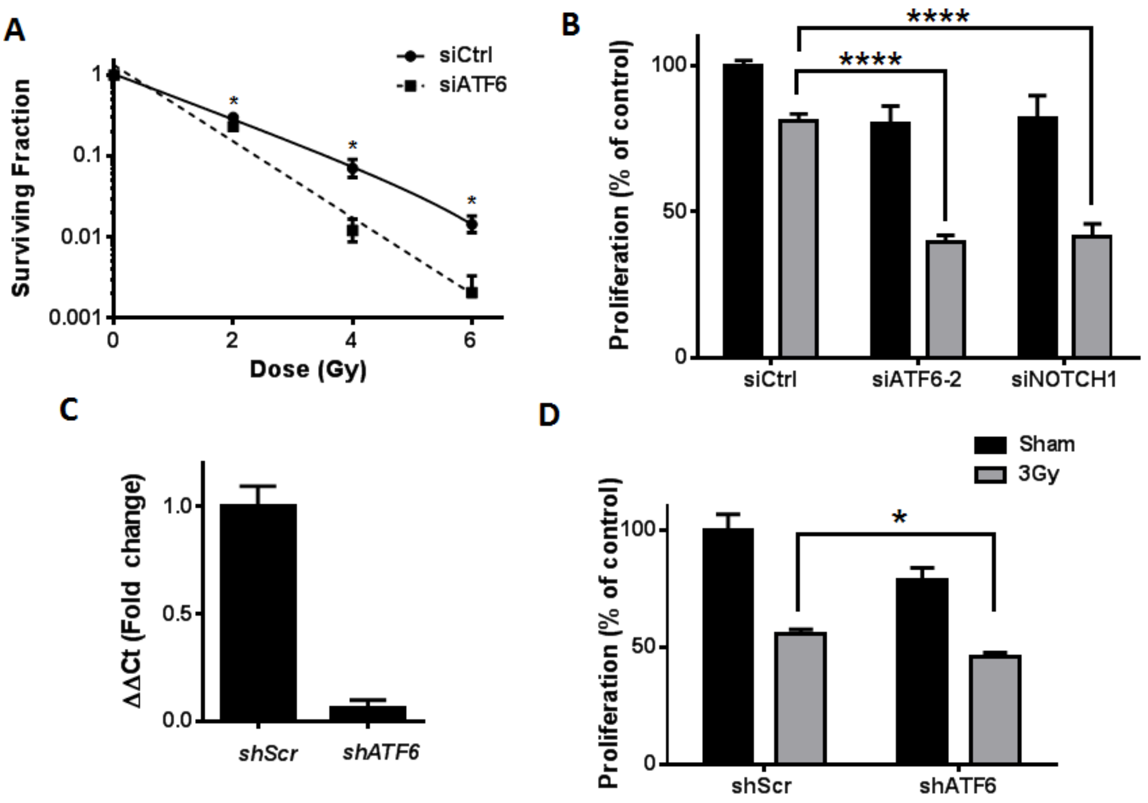

Supplementary Figure 3: Knockdown of ATF6 attenuates proliferation and clonogenic survival. (A) Clonogenic assay of D54 cells treated with siRNA targeting ATF6. Cells were irradiated with 0, 2, 4 or 6 Gy IR 48h after transfection, and colonies comprising of at least 50 cells were counted 7-10 days after irradiation. (B) Proliferation assays of D54. Cells were treated with siRNA targeting ATF6 or NOTCH1 for 48h prior to irradiation with 3 Gy, and proliferation was determined using a colorimetric cell proliferation assay 96h after irradiation. (C) Quantitative RT-PCR analysis of ATF6 expression in D54 after shRNA-mediated knockdown of ATF6. (D) Proliferation assays of D54. D54 cells were transduced with shRNA targeting ATF6 and treated with 3 Gy. Proliferation was determined using a colorimetric cell proliferation assay 96h after irradiation. In all graphs, data shown are the Means ±SD (n = 5).

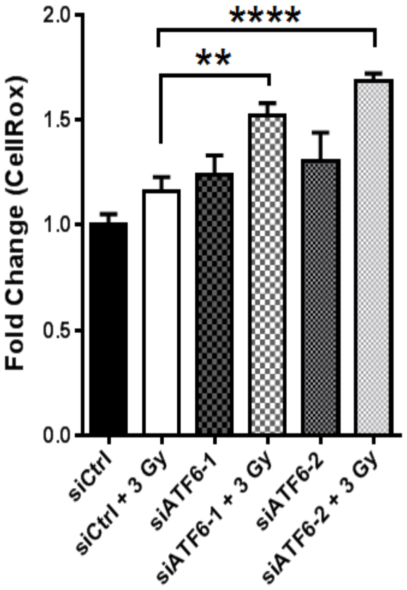

Supplementary Figure 4: Knockdown of ATF6 enhances radiation-induced ROS. CellRox assay of D54. D54 cells were treated with siRNA targeting ATF6 for 48h prior to irradiation with 3 Gy. Cells were stained with CellRox Deep-Red 96h after irradiation, and analyzed by flow cytometry. Data shown are the Means ±SD (*n* = 3). ** *P* <0.01, **** *P*<0.0001.

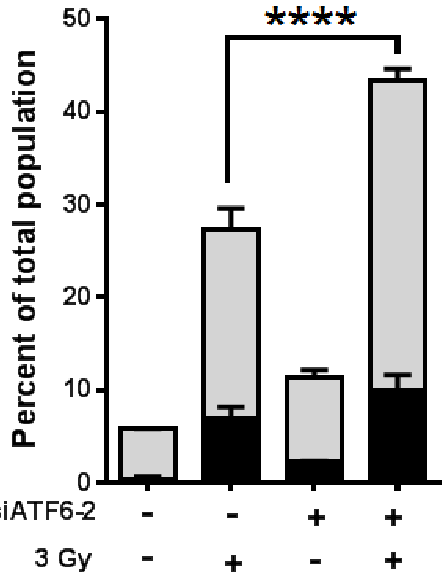

Supplementary Figure 5: Knockdown of ATF6 with another siRNA sequence results in sensitization to IR. Apoptosis assay of D54. D54 cells were treated with siRNA targeting ATF6 for 48h prior to irradiation with 3 Gy. Cells were stained with Annexin V and PI 96h after irradiation, and analyzed by flow cytometry. Data shown are the Means ±SD (n = 3). **** P<0.0001.

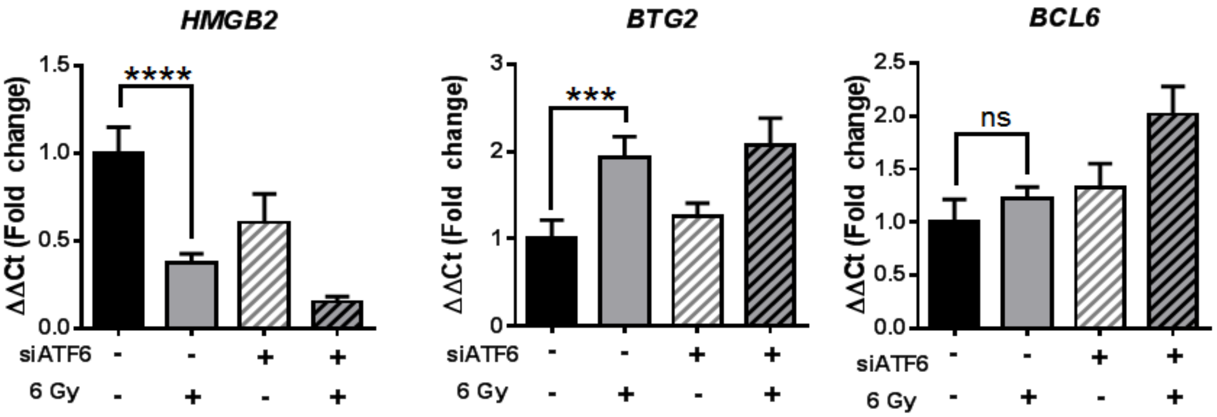

Supplementary Figure 6: Expression of genes bearing putative ATF6 binding sites in irradiated D54. Quantitative RT-PCR analysis of *HMGB2*, *BTG2*, and *BCL6* in D54. D54 cells were treated with ATF6 siRNA for 48h prior to irradiation with 6 Gy. ns = not significant, *** *P*<0.001, **** *P*<0.0001.

Supplemental Table 1.

Quantitative PCR Primers

Gene	Forward	Reverse
ACTIN	CATGTACGTTGCTATCCAGGC	CTCCTTAATGTCACGCACGAT
ATF4	GTTCTCCAGCGACAAGGCTA	ATCCTCCTTGCTGTTGTTGG
ATF6	TTATCAGCATACAGCCTGCG	CTTGGGACTTTGAGCCTCTG
BCL2	GGTGGGGTCATGTGTGTGG	CGGTTCAGGTACTCAGTCATCC
BCL6	GTTGTGGACACTTGCCGGAA	CTCTTCACGAGGAGGCTTGAT
BCLXL	GAGCTGGTGGTTGACTTTCTC	TCCATCTCCGATTCAGTCCCT
BTG2	ACCACTGGTTTCCCGAAAAG	CTGGCTGAGTCCGATCTGG
EDEM	CAAGTGTGGGTACGCCACG	AAAGAAGCTCTCCATCCGGTC
GADD34	ATGATGGCATGTATGGTGAGC	AACCTTGCAGTGTCCTTATCAG
GAPDH	GGAGCGAGATCCCTCCAAAAT	GGCTGTTGTCATACTTCTCATGG
GRP78	CATCACGCCGTCCTATGTCG	CGTCAAAGACCGTGTTCTCG
HERPUD	AACGGCATGTTTTGCATCTG	GGGGAAGAAAGGTTCCGAAG
HMGB2	CCGGACTCTTCCGTCAATTTC	GTCATAGCGAGCTTTGTCACT
HYOU1	GCAGACCTGTTGGCACTGAG	TCACGATCACCGGTGTTTTC
MCL1	GTAATAACACCAGTACGGACGG	CCACAAACCCATCCTTGGAAG
NOTCH1	GAGGCGTGGCAGACTATGC	CTTGTACTCCGTCAGCGTGA
SURVIVIN	AGGACCACCGCATCTCTACAT	AAGTCTGGCTCGTTCTCAGTG
XBP1-S	CTGAGTCCGAATCAGGTGCAG	ATCCATGGGGAGATGTTCTGG
